# Stable factor structure of the Edinburgh Postnatal Depression Scale during the whole peripartum period: Results from a Japanese prospective cohort study

**DOI:** 10.1038/s41598-018-36101-z

**Published:** 2018-12-05

**Authors:** Chika Kubota, Toshiya Inada, Yukako Nakamura, Tomoko Shiino, Masahiko Ando, Branko Aleksic, Aya Yamauchi, Mako Morikawa, Takashi Okada, Masako Ohara, Maya Sato, Satomi Murase, Setsuko Goto, Atsuko Kanai, Norio Ozaki

**Affiliations:** 1 National Center of Neurology and Psychiatry, Kodaira, Tokyo, Japan; 2 Department of Psychiatry, Nagoya University Graduate School of Medicine, Nagoya, Aichi Japan; 3 Department of Psychiatry and Psychobiology, Nagoya University Graduate School of Medicine, Nagoya, Aichi Japan; 4 Center for Advanced Medicine and Clinical Research, Nagoya University Graduate School of Medicine, Nagoya, Aichi Japan; 5 Liaison Medical Marunouchi, Nagoya, Aichi Japan; 6 Goto Setsuko Ladies Clinic, Nagoya, Aichi Japan; 7 Graduate School of Education and Human Development, Nagoya University, Nagoya, Aichi Japan

## Abstract

Early detection of perinatal depression is an urgent issue. Our study aimed to examine the construct validity and factor structure of the Japanese version of the Edinburgh Postnatal Depression Scale (EPDS) from a prospective cohort study from pregnancy to postpartum. A total of 1075 women completed all items of the EPDS at four time points: early pregnancy, late pregnancy, 5 days postpartum and 1 month postpartum. The participants were randomly divided into two sample sets. The first sample set (n = 304) was used for exploratory factor analysis, and the second sample set (n = 771) was used for confirmatory factor analysis. As a result, the Cronbach’s alpha coefficients of the EPDS items were 0.762, 0.740, 0.765 and 0.772 at the four time points. From the confirmatory factor analysis of the EPDS in a sample set of Japanese women from pregnancy to postpartum, the following three factors were detected: depression (items 7, 9), anxiety (items 4, 5) and anhedonia (items 1, 2). In conclusion, the EPDS is a useful rating scale, and its factor structure is consistently stable during the whole peripartum period.

## Introduction

Early detection of peripartum depression is an urgent issue. Peripartum depression not only decreases maternal quality of life, but also negatively affects maternal physical health^1^ and increases the risk of suicide^2^. Peripartum depression has been reported to be associated with preterm birth^3^, as well as the birth weight^3^ and cognitive^4,5^ and emotional^5^ development of the child.

The Edinburgh Postnatal Depression Scale (EPDS), a 10-item self-administered questionnaire, was originally developed to screen for postpartum depression^6^. In recent years, the EPDS has been validated for use during pregnancy for early detection of perinatal depression.

The initial symptoms of perinatal depression that extend from pregnancy to postpartum can be clarified by determining the factor structure of the EPDS during pregnancy. Therefore, factor analyses of the EPDS for this period have been investigated in many countries. However, most of these studies were cross-sectional, and only examined a single time point. Moreover, confirmatory factor analysis has scarcely been performed. Our recent study revealed the factor structure of the Japanese version of the EPDS, but only at 1 month postpartum^7^.

According to previous studies, the factor structure of the EPDS differs depending on the peripartum time points. For example, Jomeen & Martin *et al*. showed the factor structure of the original version of the EPDS differs depending on the weeks of gestation. Item 8 “I have felt sad or miserable” was included in an anhedonia factor at 14 weeks of gestation^8^, whereas it was included in an anxiety factor at 27–40 weeks of gestation^9^. Cunningham *et al*. reported that the EPDS has a three-factor structure of anhedonia, anxiety and depression in outpatients, but only a two-factor structure of depression and the other in hospitalized patients^10^. A variety of previous results of the factor structure suggest that the symptomatologic features may differ depending on the peripartum time points.

Factor structure analyses of the EPDS examined at various peripartum time points have shown a variety of factor structures. However, there have been few longitudinal studies examining the factor structure of the EPDS at several time points throughout the peripartum period. The aim of the present prospective cohort study using the identical sample set was to examine whether the factor structure of the EPDS varies depending on the peripartum time point.

## Results

### Characteristics of participants

Participants were evaluated using the Japanese version of the EPDS at the following four time points: early pregnancy (25.1 weeks gestation, standard deviation [SD] 7.1 weeks), late pregnancy (36.2 weeks gestation, SD 1.0 weeks), 5 days postpartum, and 1 month postpartum (32.5 days postpartum SD 8.1 days). The mean age of the participants was 32.3 years (SD 4.7 years). The mean years of education was 14.7 (SD 1.8 years). Regarding the number of births, the rate of nulliparous, primiparous, and those who had given birth twice or three times was 75.0%, 19.6%, 4.9% and 0.6%, respectively. The annual mean household income was 55,300 (SD 21,000) US dollars. Regarding employment status, 37.5% of participants were homemakers, while 62.5% were full- or part-time workers. A total of 1075 out of 1240 participants completed all items of the EPDS.

### Exploratory factor analysis (EFA)

The first sample set (a total of 30%, n = 304) was used for EFA. The results of The Kaiser-Meyer-Olkin (KMO) Test and EFAs at early pregnancy, late pregnancy, 5 days postpartum, and 1 month postpartum were 8.46, 8.68, 8.79, and 8.82, respectively. The KMO Measure of Sampling Adequacy at each time point indicated a reasonable value for factor analysis. The number of factors was examined from the scree plot, and the three-factor structure was considered in EFAs. The factor correlations were considered to exist, and oblique rotation was selected. Correlation between factor I-II were 0.611, 0.431, 0.676 and 0.711, II-III were 0.511, 0.702, 0.559 and 0.587, and III-I were 0.393, 0.591, 0.634 and 0.582 at early pregnancy, late pregnancy, 5 days postpartum, and 1 month postpartum, respectively. Items with factor loading exceeding 1 were found due to high correlation between three factors, and EPDS items with the highest factor coefficient level among the three factors were defined in the same factor, as shown in Table 1.

**Table 1:** EFAs of the EPDS at each time point (N = 304, maximum-likelihood estimation, promax rotation; items with the highest factor coefficient level among the three factors were defined in the same factor).

Early Pregnancy	I	II	III
EPDS1	0.198	0.007	**0.553**
EPDS2	−0.060	0.018	**1.025**
EPDS3	0.012	**0.636**	0.015
EPDS4	0.016	**0.784**	−0.014
EPDS5	0.001	**0.774**	−0.068
EPDS6	−0.074	**0.466**	0.169
EPDS7	**0.544**	0.021	0.201
EPDS8	**0.599**	0.310	0.001
EPDS9	**1.021**	−0.181	−0.002
EPDS10	**0.625**	0.133	−0.052
Variance(%)	**68.341**		
**Late Pregnancy**	**I**	**II**	**III**
EPDS1	−0.158	**0.621**	0.327
EPDS2	0.092	**1.035**	−0.134
EPDS3	**0.767**	−0.052	−0.014
EPDS4	**0.756**	0.027	−0.013
EPDS5	**0.638**	−0.003	0.052
EPDS6	**0.281**	0.180	0.048
EPDS7	0.211	0.123	**0.479**
EPDS8	0.382	−0.045	**0.524**
EPDS9	−0.057	0.007	**0.854**
EPDS10	0.041	0.017	**0.514**
Variance(%)	**66.910**		
**5 Days Postpartum**	**I**	**II**	**III**
EPDS1	0.133	0.005	**0.733**
EPDS2	−0.043	0.073	**0.860**
EPDS3	**0.736**	0.109	−0.098
EPDS4	**0.749**	−0.084	0.084
EPDS5	**0.658**	−0.023	0.061
EPDS6	**0.459**	−0.057	0.051
EPDS7	0.153	**0.594**	0.069
EPDS8	0.389	**0.555**	−0.098
EPDS9	−0.201	**0.980**	0.061
EPDS10	0.212	**0.256**	0.113
Variance(%)	**66.991**		
**1 Month Postpartum**	**I**	**II**	**III**
EPDS1	−0.041	−0.083	**1.068**
EPDS2	0.066	0.097	**0.673**
EPDS3	**0.743**	0.042	0.029
EPDS4	**0.941**	−0.105	−0.040
EPDS5	**0.725**	0.070	−0.035
EPDS6	**0.367**	0.053	0.153
EPDS7	0.121	**0.494**	0.166
EPDS8	0.380	**0.433**	0.060
EPDS9	−0.108	**0.976**	−0.034
EPDS10	0.056	**0.600**	−0.060
Variance(%)	**70.165**		

### Confirmatory factor analysis (CFA)

The other sample set (a total of 70%, n = 771) was used for CFA. The models obtained in EFAs were verified in CFAs, as shown in Table 2.

**Table 2:** CFAs of the EPDS and the best model selected at each time point (N = 771).

Models		Chi-squared/df	CFI	RMSEA	AIC
Early pregnancy	F1: 7–10 F2: 3–6 F3: 1, 2	103.421/32 = 3.232	0.973	0.027	169.041
	F1: 4, 5 F2: 7, 9 F3: 1, 2	7.694/6 = 1.282	0.999	0.01	**49.694**
Late pregnancy	F1: 3–6 F2: 1,2 F3: 7–10	95.847/32 = 2.995	0.975	0.025	161.847
	F1: 4, 5 F2: 7, 9 F3: 1, 2	19.824/6 = 3.304	0.99	0.027	**61.824**
5 days postpartum	F1: 3–6 F2: 7–10 F3: 1,2	144.180/32 = 4.506	0.964	0.034	210.18
	F1: 4, 5 F2: 7, 9 F3: 1, 2	8.151/6 = 1.356	0.999	0.011	**50.151**
1 month postpartum	F1: 3–6 F2: 7–10 F3: 1,2	142.232/32 = 4.507	0.961	0.033	208.232
	F1: 4, 5 F2: 7, 9 F3: 1, 2	6.791/6 = 1.132	0.999	0.007	**48.791**

The measurement invariance of the three-factor structure model was examined using the multiple group structural equation modeling method. The items with an absolute Z-score of 1.96 or higher were eliminated at many time points in order as follows (T1: early pregnancy, T2: late pregnancy, T3: 5 days postpartum, and T4: 1 month postpartum); firstly, item 8 (T1 vs T2: −3.736, T1 vs T3: −6.904, T1 vs T4: −3.469, T2 vs T3: −3.453 and T3 vs T4: 3.176) and item 10 (T1 vs T2: −3.287, T1 vs T3: −2.633, T2 vs T4: 3.431 and T3 vs T4: 2.823); secondly, item 6 (T1 vs T3: 3.575, T1 vs T4: 3.143, T2 vs T3: 2.767 and T2 vs T4: 2.366); and thirdly, item 3 (T1 vs T4: 2.201). Finally, the three-factor model (F1: 7, 9; F2: 4, 5; F3: 1, 2) was identified in which all Z-scores were less than 1.96. We defined the best three-factor model as follows; anxiety factor (items 4 and 5), depression factor (items 7 and 9), and anhedonia factor (items 1 and 2), respectively.

**Figure 1:**
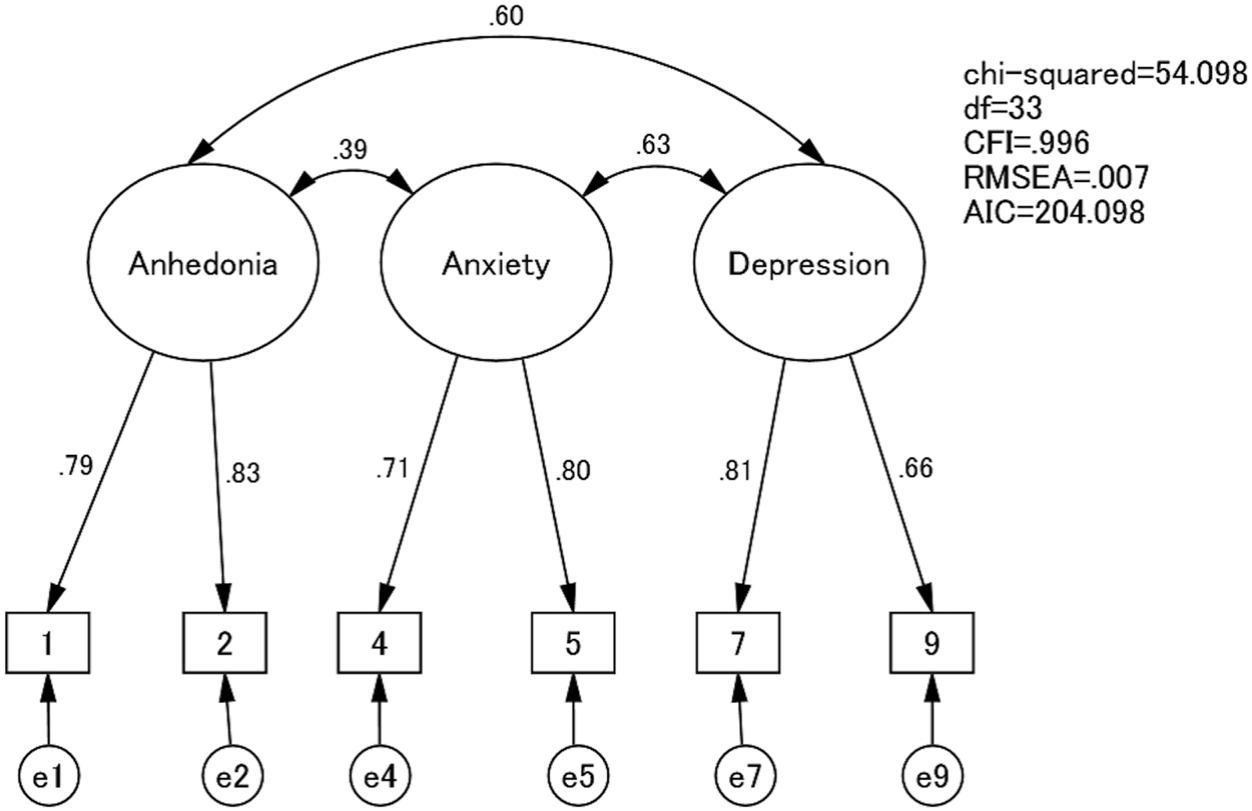
The path diagram for the best-fit model in CFAs.

The goodness of fit assuming the stable path coefficient was more satisfactory than the one free from constraint, and the robust factor structure model was confirmed as follows: the measurement invariance model (CMIN/df = 54.098/33, CFI = 0.996, RMSEA = 0.007, AIC = 204.998, and p = 0.235) and the inhabited model (CMIN/df = 42.461/24, CFI = 0.997, RMSEA = 0.008, AIC = 210.461). The goodness of fit of this model at each point is also shown in Table 2, and the path diagram for the measurement invariance model is shown in Fig. 1.

### Reliability

The Cronbach’s alpha coefficients of all 10 items of the EPDS were 0.762, 0.740, 0.765 and 0.772 at early pregnancy, late pregnancy, 5 days postpartum and 1 month postpartum.

### EPDS and its subscale scores

The rates of participants with a score greater than or equal to the cut-off point at early pregnancy, late pregnancy, 5 days postpartum and 1 month postpartum were 7.1%, 4.9%, 19.9% and 21.5%, respectively. The mean score and range for each factor are shown in Table 3.

**Table 3:** Mean EPDS scores at four time points.

N = 1075	Early pregnancy	Late pregnancy	5 days postpartum	1 month postpartum
Anxiety	1.7	1.54	1.35	1.48
(SD, range)	(1.63, 0–6)	(1.51, 0–6)	(1.58, 0–6)	(1.61, 0–6)
Depression	0.48	0.54	0.55	0.5
	(0.95, 0–6)	(1.00, 0–6)	(1.03, 0–6)	(1.00, 0–6)
Anhedonia	0.24	0.22	0.35	0.4
	(0.72, 0–6)	(0.72, 0–6)	(0.84, 0–6)	(0.86, 0–6)
6 items (1,2,4,5,7 and 9) of EPDS	2.42	2.3	2.25	2.38
	(2.63, 0–17)	(2.63, 0–18)	(2.88, 0–17)	(2.84, 0–16)
10 items of EPDS	4.77	4.29	4.92	5.26
	(4.45, 0–29)	(4.34, 0–30)	(4.85, 0–29)	(4.88, 0–27)

## Discussion

The reliability and construct validity of the EPDS during the periods from pregnancy to postpartum were established in our prospective cohort study including a total of 1075 participants. The three-factor structure model of the Japanese version of the EPDS was consistently observed at each of the time points from pregnancy to postpartum. The three factors detected were anxiety, depression and anhedonia.

The factor structure of the Japanese version of the EPDS at postpartum was specified in our study in 2014^7^; however, its factor structure during pregnancy remained unclear. Consequently, it was not appropriate to apply the postpartum factor structure to the one during pregnancy. However, with the larger sample size, the present study clearly demonstrated that the Japanese version of the EPDS had a robust factor structure with excellent reliability, validity, and stability from pregnancy to postpartum. Based on this result, the progress and changes of symptoms indicated by factors throughout the perinatal period can be examined.

Various factor structures were found at different peripartum time points in the cross-sectional studies summarized in Table 4. It is unclear whether these differences in the factor structure stem from the time points examined or racial differences. Chiu *et al*. reported that the factor structure of the EPDS was stable across Hispanics, whites and African Americans in the postpartum period^11^. Our three-factor model in the postpartum period is similar to that of other studies, as well. King *et al*.^12^ and Cunningham *et al*.^10^ also reported a three-factor model containing depression, anhedonia and anxiety, despite these studies being conducted separately in Australia and the United States.

**Table 4:** Summary of previous cross-sectional studies using confirmatory factor analysis of EPDS.

First author, Published year	Country	Version	N	Time period	Factor structure
Toreki, A., 2013^20^	Hungary	Hungarian	219	Pregnancy	F1: 2, 4, 5, 6, 10
				12 weeks	F2: 3, 8, 9
					F3: 1, 7
Jomeen, J., 2005^8^	UK	English	101	Pregnancy	F1: 1, 2, 8
				14 weeks	F2: 3, 4, 5
Zhong, Q., 2014^21^	Peru	Spanish	1517	Pregnancy	F1: 3–10
				0–16 weeks	F2: 1, 2
Jomeen, J., 2007^9^	UK	English	148	Pregnancy	F1: 1, 2
				27–40 weeks	F2: 3, 4, 5, 8
Cunningham, N.K., 2015^10^	Australia	English	875	Pregnancy	Outpatient care
				0–40 weeks	F1: 1, 2
				Postpartum	F2: 3, 4, 5
				0–12 months	F3: 6–10
					Hospitalization
					F1: 1, 2, 3, 6–10
					F2: 4, 5
King, P. A., 2012^12^	US	English	169	Postpartum	F1: 7–10
				0–12 months	F2: 1, 2
					F3: 3, 4, 5
Phillips, J., 2009^22^	Australia	English	309	Postpartum	F1: 1, 2, 6–10
				0–12 months	F2: 3, 4, 5
Bina, R. & Harringtom, D., 2016^23^	Israel	Hebrew	969	Postpartum	F1: 1, 2, 7–10
				6 weeks	F2: 3, 4, 5
Gollan, J. K., 2017^24^	US	English	15172	Postpartum	F1: 1, 2, 6–10
				4–6 weeks	
Chiu, Y.-H. M., 2017^11^	US	English	515	Postpartum	F1: 7, 8, 9
				6 months	F2: 3–6
					F3: 1, 2

UK, United Kingdom; US, United States.

Regarding the longitudinal prospective study of the factor structure of the EPDS throughout the peripartum period, Coates *et al*.^13^ examined the factor structure of the EPDS in pregnancy and postpartum at four time points, i.e. at 18 and 32 weeks gestation; and at 8 weeks and 8 months postpartum, in a large population-based sample (n = 11,195–12,166) and found that the EPDS appears to measure three related factors of depression, anhedonia, and anxiety and has a stable structure in pregnancy and the first postpartum year^13^. Our present study replicated the findings by Coates *et al*.^13^ of a robust factor structure observed at different time points during the peripartum period, regardless of the different language and culture between Japan and the UK.

The EPDS results and its subscale scores highlight some important findings. Firstly, the rate of participants with an EPDS score higher than or equal to the cut-off point differed between pregnancy and postpartum. This difference may be due to the difference in the criteria of the cut-off scores between pregnancy and postpartum, as reported in previous Japanese studies^14,15^. Secondly, the highest score was observed in the items of the anxiety factor, when compared to those of the depression and anhedonia factors throughout the peripartum period. This suggests that perinatal depression consists of mainly anxiety symptoms. The current DSM-5 (DSM: Diagnostic and Statistical Manual of Mental Disorders) diagnosis of major depressive disorders, which does not include the anxiety symptoms, may not fit the current factor structure of perinatal depression. Further research is needed to elucidate whether perinatal depression could be separated from major depression as defined in DSM-5.

A major limitation of the present study is possible sampling bias The facilities participating in the present study were restricted to the urban area around Nagoya, Japan, and high-risk pregnancies often present at university hospitals. Moreover, participants who were physically or mentally impaired may have dropped out of the study because of difficulty responding to the questionnaire during the whole peripartum period. However, our demographic data are comparable to the data of national census research reported by the Ministry of Health, Labour and Welfare in 2014–2015, so our sample likely represents the population of peripartum women in Japan.

In the present prospective cohort study from pregnancy to postpartum, the reliability and factor consistency of the Japanese version of the EPDS were demonstrated. The three factors of depression, anxiety and anhedonia were detected at each of four time points in the peripartum period. Therefore, we conclude that the EPDS is a useful rating scale, and that its factor structure is consistently stable during the whole peripartum period. Further research using another randomly divided sample set is needed to confirm the validity of these results.

## Methods

### Ethical considerations

This study was approved by the Ethics Committee of the Nagoya University Graduate School of Medicine. All methods were performed in accordance with the relevant guidelines and regulations. Participants meeting the eligibility criteria were provided with an oral and written explanation of the research design and methods. At a later date, they return a set of documents on informed consent and questionnaires.

### Participants

The participants were recruited between August 2004 and June 2017 from the obstetrical outpatient care or maternity class of the following four facilities: one general hospital (Nagoya Teishin Hospital), two obstetrics and gynecology hospitals (Kaseki Hospital and Royal Bell Clinic), and one university hospital (Nagoya University Hospital). The eligibility criteria included: (1) attending one of the four facilities consecutively, (2) 20 years of age or older, and (3) ability to understand the questionnaire written in Japanese.

### Measurements

The demographic information of the participants, including age, years of education, number of births, economic status, and employment status, was obtained from the answers to the self-administered questionnaire that was completed during early pregnancy. Participants were evaluated using the Japanese version of the EPDS, established by Okano *et al*. (1996), at the following four time points: early pregnancy, late pregnancy, 5 days postpartum, and 1 month postpartum. Regarding the Japanese version of the EPDS, the cut-off point of 12/13 was proposed to indicate a possible depressive state during pregnancy^15^. Its sensitivity, specificity and positive predictive value were 90%, 92% and 55%, respectively^15^. The cut-off point of 8/9 was proposed to indicate possible postpartum depression^14^. Its sensitivity, specificity and positive predictive value were 75%, 93% and 50%, respectively^14^.

### Statistical analysis

The listwise deletion method was used for handling missing data. To examine the internal reliability of the EPDS at the individual time points and Cronbach’s alpha coefficients of all 10 items of the EPDS at each time point were calculated. Logarithmic transformation was performed for each item of the EPDS.

In the EFA, construct validity and configural invariance were examined, and in the CFA, measurement invariance was examined. These factor analyses were conducted after an assessment of specimen validity using the KMO Test^16^. A KMO value greater than 0.6 was considered to be appropriate for conducting the factor analysis. The participants were randomly divided into two sample sets. The first sample set (a total of 30%) was used for EFA, and the other sample set (a total of 70%) was used for CFA.

For EFAs, we used each EPDS item at four time points respectively. The number of factors was determined by the scree plot. Maximum likelihood estimation was used as the factor extraction method. The promax rotation was performed assuming the correlation of each factor. EPDS items with the highest factor coefficient level among the factors were defined in the same factor.

For CFAs, the models obtained in EFAs were verified. The goodness of fit of the models was assessed using the Chi-square normalized by degrees of freedom (CMIN/dF), comparative fit index (CFI)^17^, root mean square error of approximation (RMSEA)^18^ and Akaike’s Information Criterion (AIC)^19^. A CFI greater than 0.97 is considered to be good and a CFI greater than 0.95 acceptable^17^. An RMSEA less than 0.05 is considered to be good and an RMSEA less than 0.08 acceptable^18^. Lower values for CMIN/df and AIC indicate a better fit^19^. The model with the highest goodness of fit was examined using the multiple group structural equation modeling method to identify the subscale items of the factors that have measurement invariance in CFAs. The value of the path coefficient indicates the degree of influence the factor has on the subscale item. Assuming the stable path coefficient throughout four time points, the Z-score of each EPDS item was calculated. The Z-score is a statistical value for assessing the difference in the path coefficient at each time point. The items with an absolute Z-score of 1.96 or higher were eliminated because they did not exhibit measurement invariance. Based on these results, the best factor structure model having configural and measurement invariance throughout four time points was identified.

The data were analyzed using IBM SPSS Statistics version 24.0 and IBM Amos version 24.0 (IBM Japan, Tokyo, Japan).
